# Zero-waste multistage utilization of dandelion root

**DOI:** 10.3389/fchem.2024.1457813

**Published:** 2024-08-23

**Authors:** Yongwei Fang, Aimin He, Weihua Chen, Xiaohui Jia, Mingqin Zhao, Miao Lai, Hong Zhang

**Affiliations:** ^1^ College of Tobacco Science, Henan Agricultural University, Zhengzhou, Henan, China; ^2^ China Tobacco Hebei Industrial Co., Ltd., Shijiazhuang, Hebei, China

**Keywords:** dandelion root, biomass recycling, porous carbon, zero-waste, flavors and fragrances

## Abstract

**Introduction:**

In the fragrance and perfume industry, the controlled release of fragrances are crucial factors that contribute to consumer appeal and product quality enhancement. In this study, various aromatic active substances were extracted from dandelion root (DR), which was subsequently calcined to produce high-performance porous biochar material.

**Methods:**

The dandelion root biochar (DRB) material was identified as promising adsorbents for the controlled release of fragrances. Furfuryl alcohol was chosen as the model fragrance for inclusion and controlled release studies.

**Results and discussion:**

The DRB exhibited a substantial specific surface area of 991.89 m^2^/g, facilitating efficient storage and controlled release capabilities. Additionally, the DRB’s high stability and porous nature facilitated rapid collection and efficient recyclability. This research significantly contributes to the development of a sustainable, zero-waste multistage utilization strategy for dandelion roots, indicating a potential applications in the food flavoring industry and environmental conservations.

## 1 Introduction

Fragrances are widely used in various industries, including textiles, leather, paper, cosmetics, and agriculture, due to their advantages of delightful scents, antimicrobial activity, calming effects, and therapeutic benefits (Indriyani et al., 2024; Liu et al., 2024; Wu et al., 2024). Ensuring a steady and enduring release of aromatic molecules could enrich customer experiences and amplify their positive impacts. However, fragrances are often highly volatile, unstable, and prone to oxidation, which poses challenges during storage and application. Therefore, it is essential to develope spice delivery materials that can preserve these molecules and maintain a long-lasting aroma.

With the development of science and technology, diverse adaptable nanomaterials have been adopted for preserving and regulating the emission of aromatic molecules, including polymeric, inorganic, and composite nanocapsules (Yang et al., 2023; Zhang et al., 2019; Souza et al., 2019). Typically, aromatic molecules are introduced into these nanomaterials through covalent linkage or physical entrapment facilitated by hydrophobic interactions and electrostatic attraction (Xiao et al., 2022; Son et al., 2022; Han et al., 2021; Zhang and Ye., 2021). Nevertheless, the applicability of this technique is significantly constrained, as it is only compatible with fragrances bearing specific reactive groups, such as aldehydes. Microencapsulation emerges as an efficient physical entrapment technology capable of encapsulating a wide array of volatile compounds within microcapsules spanning from nanometers to micrometers (Xiao et al., 2022; Xu et al., 2021; Zhao et al., 2016). The dispersal characteristics of the encapsulated volatile molecules are influenced by the thickness of the shell and the permeability of its structure. Besides, polymeric microcapsules inherently exhibit poor colloidal stability in liquids, and poor control ability of particle size, distribution, and shape (Foroutani et al., 2024; Pellicer et al., 2018; Song and Chen., 2018). However, inorganic porous materialscharacterized by their high structural stability, expansive surface area, and adjustable pore size, offer a solution to these limitations. Reports indicate that these porous spheres extend the duration of fragrance release (Tang et al., 2018; Li et al., 2018a).

In this work, we adopted a multistage utilization approach for biomass feedstock to promote waste recovery and economic recycling through an efficient and friendly framework. To demonstrate zero-waste concept, biologically active constituents firstly extracted from dandelion root, which commonly applied to chemical raw materials, biomedical agents, and food additives. Then, DRB was synthesized from dandelion root residue and then evaluated for its efficacy in the encapsulation and release of extracted fragrance. We found that DRB owning a rich spatial void structure, which could enhance their encapsulation and improve their controlled release profile. This study represents full use of dandelion root, and DRB has ability to regulate the release profile of fragrant molecules to meet the requirement of on-demand release.

## 2 Materials and methods

### 2.1 Chemicals

Dandelion root was collected from Changbai Mountainin Jilin Province. Reagents used included 1,1-diphenyl-2-pyridine hydrazine (DPPH), ascorbic acid (AR), salicylic acid (AR), ferrous sulfate (98%), hydrogen peroxide (30 wt% in H_2_O), anhydrous ethanol (AR), nitric acid (AR, 65%), potassium hydroxide (AR), sulfuric acid (AR, 98%), furfuryl alcohol (AR, 98%), hydrochloric acid (AR, 36%), ultrapure water, and nitrogen. All other chemicals were of analytical grade and used directly without further purication.

### 2.2 Biochar characterization

The surface morphology of the biochar was examined using a scanning electron microscope (ZEISS Gemini Sigma 300, German). The aromatic adsorbents were analyzed with a X-ray diffractometer (Malvern Panalytical Empyrean, Uinted Kingdom). Structural parameters of the adsorbents were determined using a Automatic Specific Surface and Porosity Analyzer (Micromeritics ASAP 2460, United States). The spices and adsorbents were analyzed using a Fourier-transform infrared spectrometer (Thermo Scientific Nicolet iS20, United States). The thermal stability of the samples was assessed using a simultaneous thermal analyzer (HITACHI STA200, Japan). Absorbance measurements were conducted using a microplate reader (SPARK 10M, Austria) and a spectrophotometer (Shimadzu 180 UV, Japan).

### 2.3 Sample extraction

Dandelion root was pre-treated with ultrapure water, dried at 60°C until it reached a constant weight, and then crushed in a grinder. The dried dandelion root was extracted with anhydrous ethanol at a solid-to-liquid ratio of 1:20. The sample mixture was distilled in a water bath for 4 h, followed by rapid vacuum pump filtration. The anhydrous ethanol filtrate was concentrated to 20 mL using a vacuum rotary evaporator.

### 2.4 Compositional analysis of dandelion root extract by GC-MS

Chromatographic column: HP-5 column; carrier gas: helium; column flow rate: 1 mL/min; injection volume: 1.0 μL; inlet temperature: 270°C. Programmed temperature increase conditions: initial temperature 100°C, hold for 2 min, increase to 270°C at 5°C/min, hold for 10 min. Ionization source: EI; Ionization energy: 70 eV; interface temperature: 280°C; ion source temperature: 200°C; scanning range: m/z 35–700; standard library: NIST 17 Spectral Library.

### 2.5 Analysis of antioxidant activity of dandelion root extracts

DPPH solution with a concentration of 0.1 mmol/L was configured according to the method of Cai (Cai et al., 2019) and others with slight modifications. Take 0.5 mL of different concentrations of the samples respectively (1, 5, 10, 15, 20, 25 mg/mL), add 2 mL of DPPH solution, then add 1.5 mL of anhydrous ethanol, mix well, and shake at room temperature and avoid light for 30 min, use the microplate reader to determine the absorbance value at 517 nm. In the control group, 3.5 mL of anhydrous ethanol was added to 0.5 mL of sample solution, and the absorbance value was measured at 517 nm. In the blank group, 2 mL of anhydrous ethanol was added to 2 mL of DPPH-ethanol solution at a concentration of 0.1 mmol/L instead of the sample solution, and the absorbance value was measured at 517 nm. The DPPH radical scavenging rate was calculated according to Formula 1:

$$K\%=\left(1-\frac{A_{2}-A_{1}}{A_{3}}\right)\times 100\%$$ (1)

In Formula 1, A_1_ is the absorbance of the sample solution; A_2_ is the absorbance of the control group; A_3_ is the absorbance of the blank group.

Briefly, 1 mL of 9 mmol/L FeSO_4_ solution, 1 mL of 9 mmol/L H_2_O_2_ solution, 1 mL of 9 mmol/L salicylic acid solution were added to 1-mL samples of different concentrations (1, 5, 10, 15, 20, 25 mg/mL), mixed, reacted at 37°C for 30 min, and the absorbance A_i_ at 562 nm was measured by using an enzyme marker. Meanwhile, the control group was used to measure the absorbance A_j_ by substituting ultrapure water for H_2_O_2_. Anhydrous ethanol was used instead of different concentrations of sample solutions to measure the absorbance A_0_. Ascorbic acid (Vc) was used as a positive control. The hydroxyl radical scavenging rate was calculated according to Formula 2:

$$K\%=\left(1-\frac{A_{I}-A_{J}}{A_{0}}\right)\times 100\%$$ (2)

In Formula 2, A_i_ is the absorbance of the sample solution; A_j_ is the absorbance of the control group; A_0_ is the absorbance of the blank group.

### 2.6 Preparation and modification of DRB

DRs were washed with ultrapure water and dried in an oven at 60°C for 8 h. These samples were then subjected to pyrolysis in a tube furnace at various temperatures x °C (x = 400, 500, 600, 700) with a heating rate of 5°C/min for varying durations y h (y = 1, 2, 4, 6, and 8). Following pyrolysis, the samples were allowed to cool to room temperature. The resulting pyrolysis products were washed with 1 M hydrochloric acid and neutralized with ultrapure water (pH 6) (Diao et al., 2021). DRB was collected on filter paper and dried at 60°C to obtain the final product.

The production process for acid-modified biochar from DRs proceeded as follows: Concentrated nitric acid was condensed and refluxed with activated carbon for 2 h at 100°C. After suction filtration and washing, the product obtained was Dandelion Roots Biochar-HNO_3_ (DRB-HNO_3_). Similarly, a mixture of concentrated nitric acid, sulfuric acid, and activated carbon was condensed and refluxed for 2 h at 100°C. After suction filtration and washing, the resulting product was Dandelion Roots Biochar-H_2_SO_4_/HNO_3_ (DRB-H_2_SO_4/_HNO_3_).

The production process for alkali-modified DRB proceeded as follows: KOH and DRs were mixed at a mass ratio of 1:1, dried in an oven at 60°C for 3 h, and then the dried solids were placed in a tube furnace under nitrogen protection for pyrolysis at high temperatures. The resulting product was Dandelion Roots Biochar-KOH (DRB-KOH).

### 2.7 Single-factor design

The adsorption properties of aromatic adsorbents were studied by ultraviolet-visible spectrophotometer. The percentage of spice adsorption is obtained by the following Formula 3, and the amount of spice adsorption can be obtained by the following Formula 4:

$$q=\frac{c_{0}-c_{1}}{c_{0}}$$ (3)

$$Q=\frac{m_{1}\times \left(c_{0}-c_{1}\right)}{m_{2}\times c_{0}}$$ (4)

In Formula 3, q is the adsorption rate of spice adsorbent; c_0_ is the concentration of spices before adsorption; c_1_ is the concentration of supernatant solution after adsorption. In Formula 4, Q is the adsorption capacity of the spice adsorbent, m_1_ is the quality of the spice, and m_2_ is the quality of the adsorbent.

The dandelion root biochar adsorbent was initially fixed at 0.1 g, and the adsorption time was set at 2 h. A specific concentration of furfuryl alcohol was utilized to prepare the spice adsorbent. Employing the adsorption rate as the evaluation index, the adsorption mode (static, ultrasonic, stirring), adsorption time (0.25, 0.5, 1, 1.5, 2 h), spice concentration (0.5, 1, 1.5, 2, 2.5 mL spice mother liquor), and adsorbent dosage (0.01, 0.03, 0.05, 0.1, 0.15, 0.2, 0.25, 0.5 g) of the spice adsorbent were optimized by conducting single-factor experiments to determine the adsorbent preparation process with the best adsorption performance.

### 2.8 Adsorption and sustained-release kinetics

The adsorption behavior of the adsorbent was evaluated through liquid-phase adsorption, and the adsorption capacity of the adsorbent was calculated at various time intervals: 1, 3, 6, 9, 12, and 24 h, respectively. To investigate the adsorption kinetic behavior of furfuryl alcohol on biochar materials, the pseudo-first-order kinetic Equation 5 (Yagub et al., 2014; Rohit et al., 2023) was employed for fitting purposes:

$$\ln\left(1-\frac{Q_{t}}{\text{Qe}}\right)=-\text{kt}$$ (5)

In Formula 5, Q_t_ and Q_e_ are the adsorption capacities (mg/g) at time t and equilibrium, respectively, while k is the adsorption rate constant (min^−1^).

Briefly, 0.1 g of the furfuryl alcohol flavor adsorbent and 1 mL of furfuryl alcohol were accurately weighed. Following the methods outlined by Zhao (Zhao et al., 2019) and other relevant protocols, respectively, place the samples under dark conditions in an oven set at room temperature 25°C and elevated temperature (80°C). Allow the samples to remain for a specified duration. Subsequently, the release rate of furfuryl alcohol was calculated using Formula 6.

$$M=M_{0}-M_{1}$$ (6)

$$W\%=\frac{M-\left(M_{1}-M_{n}\right)}{M}$$ (7)

In Formula 6, M_0_ represents the quality of the blank culture dish; M_1_ denotes the total weight of the spice adsorbent; and M indicates the weight of the spice adsorbent sample. In Formula 7, M_n_ signifies the weight of the spice adsorbent after n days, while W% represents the release rate of spices.

Four different kinetic equations were established based on the research of Cristian D, Fabricio M. B, Li M. W, et al. (Dima et al., 2016; Bezerra et al., 2018; Li et al., 2018b) to investigate the release mechanism and kinetics of aromatic adsorbents. These equations include the zero-order kinetic, first-order kinetic, Higuchi, and Peppas models, designed to fit the volatilization curves of aromatic adsorbents under various temperature conditions. The specific formulations of the four Equations 8–11 are as follows:

Zero−order kinetic model:Y=Kx (8)

$$\text{First}-\text{order kinetic model}:Y=1-\exp\left(-\text{Kx}\right)$$ (9)

$$\text{Higuchi model}:Y=Kx^{0.5}$$ (10)

$$\text{Peppas model}:Y=Kx^{n}$$ (11)

In Formulas 8–11, Y represents the release rate of spices, x represents the release time, K represents the release rate constant, and n is the diffusion index.

## 3 Results and discussion

### 3.1 Analysis of DR extracts

The volatile organic compounds in the DR extracts were analyzed using GC-MS. The results indicate that these components can be categorized into heterocyclic compounds, esters, phenols, and alkanes, which have potential applications in food processing and biomedical fields. Among the compounds detected through anhydrous ethanol extraction, eight food flavorings were identified, including 6-caprolactone, phenol, phenylacetaldehyde, resorcinol, 6-methyl-5-hepten-2-one, 4-vinyl-2-methoxyphenol, 2,6-dimethoxyphenol, and palmitic acid. Notably, phenylacetaldehyde, a cigarette additive, exhibits a robust hyacinth aroma and imparts almond and cherry flavors at low concentrations.

### 3.2 Antioxidant activity of DR extracts

The antioxidant activity of DR extracts was assessed using the DPPH free radical and hydroxyl radical scavenging assays. DPPH, being stable and easily handled, is frequently employed to gauge the efficacy of free radical scavenging antioxidants. This radical exhibits a characteristic absorbance peak at 517 nm (purple), which diminishes upon being scavenged by antioxidants (Zhang et al., 2019; Tapia-Hernández et al., 2018). Hydroxyl radicals possess one of the highest oxidative potentials among ROS. Their excessive presence can damage proteins and other vital biomolecules, such as lipids and nucleic acids, potentially leading to cancer, mutations, cytotoxicity, and chronic ailments (Tapia-Hernández et al., 2018).

As depicted in Supplementary Figure 1A, the scavenging activity of DPPH by the alcoholic extract of DRs increased with escalating concentrations, reaching 57.47% at 25 mg/mL, displaying an antioxidant capacity slightly inferior to that of the Vc positive control. Similarly, in Supplementary Figure 1B, the scavenging of OH-radicals exhibited a comparable trend, with scavenging rates ranging from 52.19% to 60.88% across concentrations spanning from 1 to 25 mg/mL, indicative of notable antioxidant performance.

### 3.3 Characterization

**FIGURE 1:**
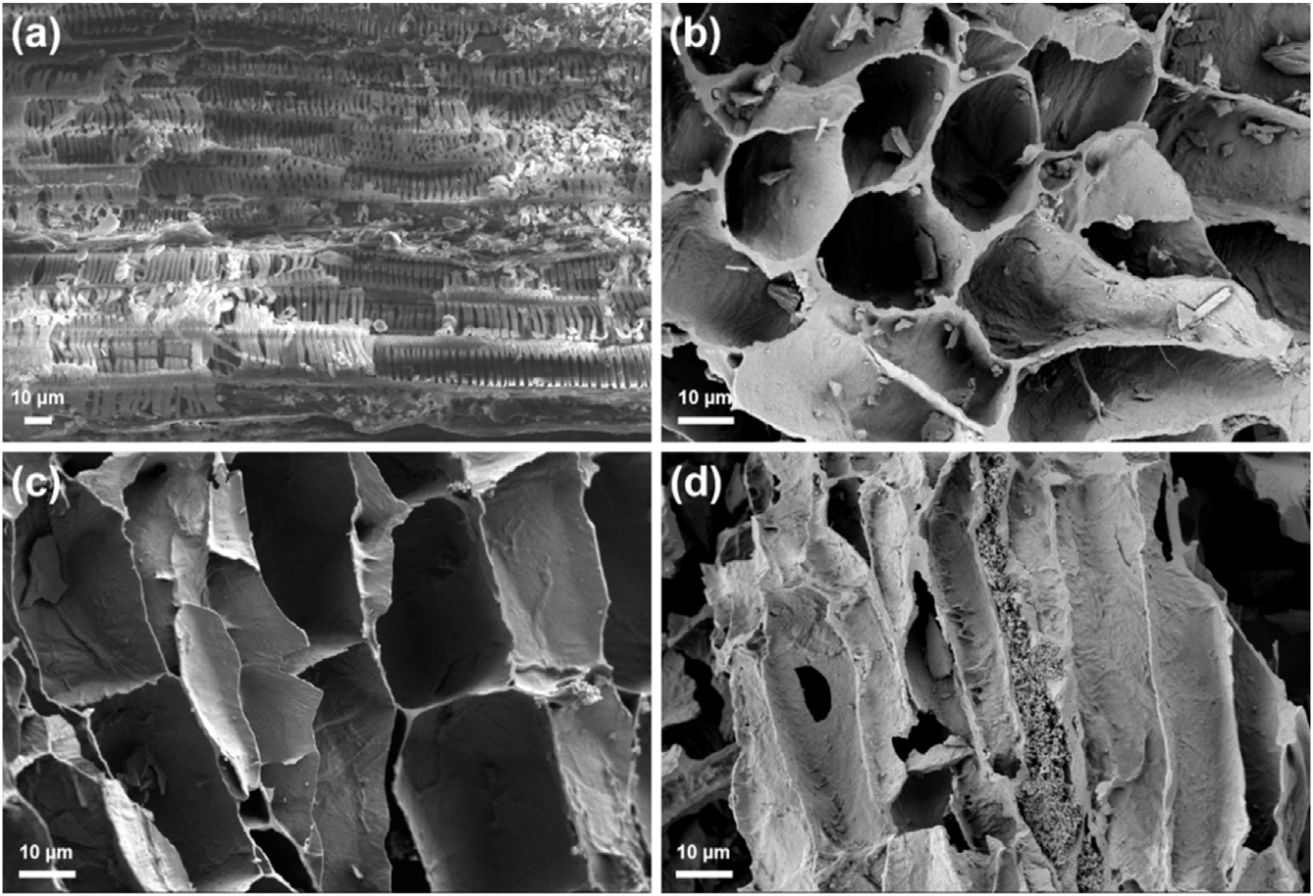
SEM of DRB **(A)**, DRB-H_2_SO_4_/HNO_3_ **(B)**, DRB-HNO_3_ **(C)** and DRB-KOH **(D)**.

The SEM findings, illustrated in Figure 1, demonstrate that the unaltered dandelion root biochar (Figure 1A) retains the inherent aqueduct structure of the plant. A lamellar structure is formed upon modification with HNO_3_ and H_2_SO_4_ (Figure 1B). Following HNO_3_ modification, the biochar treated with concentrated nitric acid exhibits substantial pore-like gaps (Figure 1C). Compared to the unaltered dandelion root biochar and acid-modified counterpart, the KOH-modified dandelion root biochar (Figure 1D) exhibits an augmentation in surface pore size and an expansion in surface area. This improvement is ascribed to the thermal decomposition of KOH at elevated temperatures, which triggers the activation and subsequent expansion of the pore structure within the biochar, as well as the disruption of its laminar surface structure.

The XRD patterns (Supplementary Figure 2) reveal a broad diffraction peak spanning from 17° to 33°, with a peak occurring approximately at 24.2°, and no distinct diffraction peak, suggesting that the material exhibits properties characteristic of amorphous carbon.

Table 1 shows the pore structure parameters of DRB before and after modification. The acid-modified DRB (DRB-HNO_3_, DRB-H_2_SO_4_/HNO_3_) exhibited a reduction in specific surface area and pore structure collapse, leading to the aggregation of multiple particles into a single particle. Consequently, there was a decrease in total pore volume and an enlargement in pore size. Conversely, the alkali-modified DRB (DRB-KOH) demonstrated a remarkable exponential increase in specific surface area, 248 times greater than before modification. Furthermore, the total pore volume expanded by approximately 231 times, while the average pore size remained relatively constant, indicating the formation of a greater number of micropores. The KOH modification and subsequent calcination induced a more porous structure and an augmentation in the number of active adsorption sites.

**TABLE 1:** Adsorbent pore structure parameters.

Adsorbent	BET surface area (m^2^/g)	Pore volume (cm^3^/g)	Pore diameter (nm)
DRB	4.4404	0.0019	1.7549
DRB-HNO_3_	1.1253	0.0015	---
DRB-H_2_SO_4_/HNO_3_	1.8459	0.0013	2.8150
DRB-KOH	991.8940	0.4402	1.7754

**FIGURE 2:**
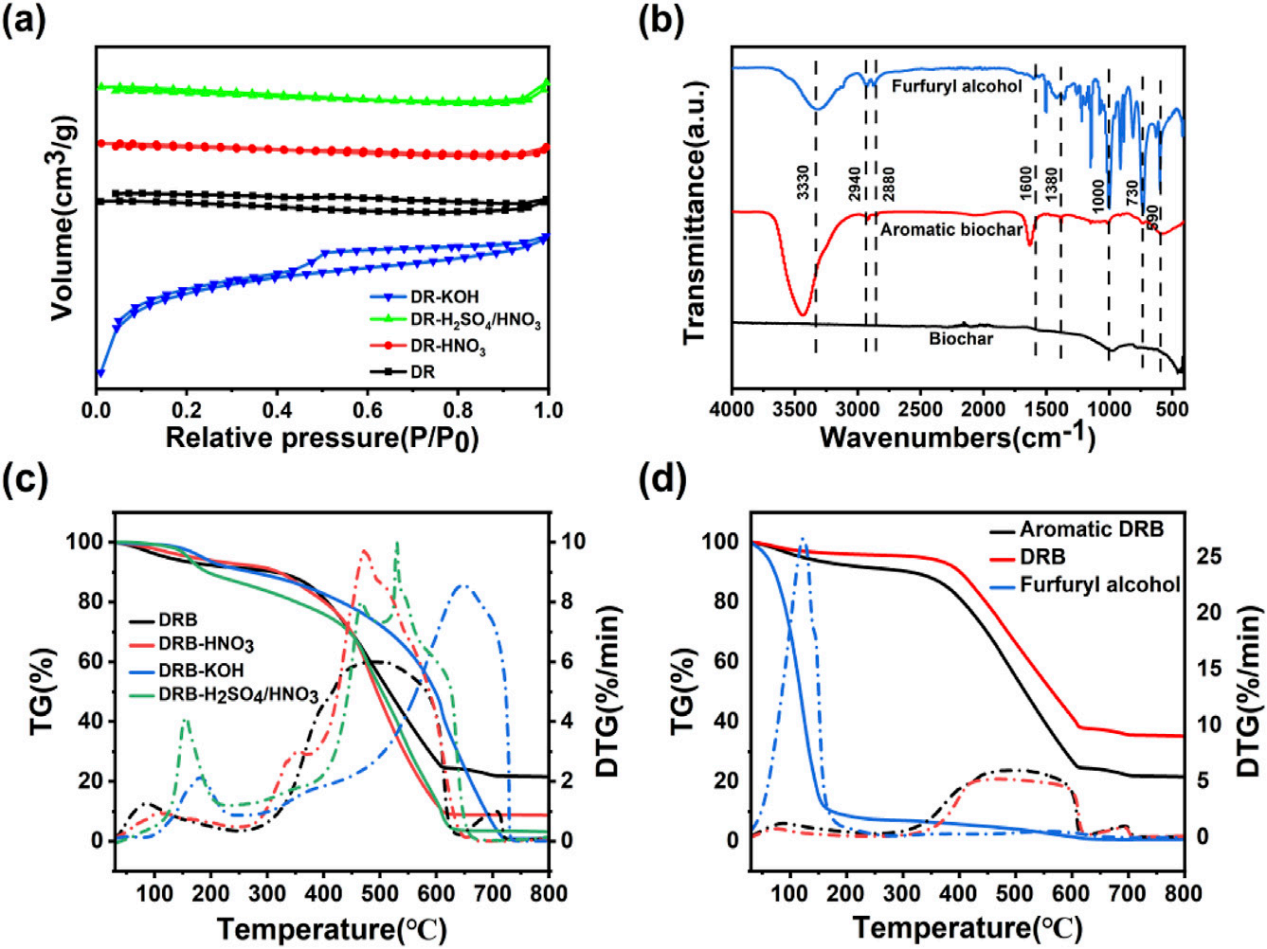
N_2_ adsorption-desorption isotherms of DRB, DRB-KOH, DRB-H_2_SO_4_/HNO_3_ and DRB-HNO_3_ **(A)**; FTIR plots of DRB-KOH biochar with furfuryl alcohol spice before and after adsorption **(B)**; TG versus DTG plots of aromatic DRB, DRB-HNO_3_, DRB-KOH, DRB-H_2_SO_4_/HNO_3_ **(C)**; TG versus DTG plots of furfuryl alcohol, aromatic DRB, DRB **(D)**.

To delve deeper into the porous properties of the samples, nitrogen (N_2_) adsorption-desorption analyses were conducted to corroborate the SEM findings and further elucidate the structure of the DRB samples. According to the classification, pores with a size φ ≤ 2 nm are considered microporous, those ranging from 2 nm to 50 nm are; classified as mesoporous, and pores with a size ≥50 nm are classified as macropores (Zhang et al., 2023). Table 1; Figure 2A show that the pores of DRB exhibit microporous characteristics.

From Figure 2A, it can be observed that all four biochars display a typical type I isothermal adsorption line, indicative of microporous filling on microporous adsorbents. The adsorption capacity of the DRB adsorbent for N_2_ gradually increases within the relative pressure range of 0 < P/P_0_ < 0.5. The overlapping adsorption/desorption curves in this region suggest the presence of small micropores adsorbed in a single layer.

DRB-KOH exhibits a distinct H4-type hysteresis loop in the range of P/P_0_ > 0.4, which is characteristic of adsorbents possessing a combination of microporous and mesoporous pores. This implies that DRB-KOH features a co-existing meso/microporous structure (Yu et al., 2024; Cuong et al., 2021). Such a meso/microporous configuration is advantageous for enhancing the diffusion and adsorption of flavor compounds on the biochar surface, thereby providing an abundant interaction channel for the sustained release of the material following spice adsorption.

As depicted in Figure 2B, several notable absorption peaks are observed. The broad peak at 3,330 cm^−1^ corresponds to the O-H stretching vibration of furfuryl spice. Additionally, an absorption peak at 2,940 cm^−1^ arises from the C-H stretching vibration, while an absorption peak at approximately 1,590 cm^−1^ signifies the vibration of the aromatic skeleton of the furan ring. Another absorption peak at around 1,600 cm^−1^ corresponds to the C=C stretching vibration of the furan ring.

Furfuryl alcohol exhibits a weak stretching vibration absorption peak at 3,300 cm^−1^. Conversely, the biochar before adsorption lacks a stretching vibration absorption peak at 3,330 cm^−1^. However, DRB-KOH adsorbed with the spice displays a stretching vibration absorption peak at 3,430 cm^−1^, slightly shifted compared to the O-H absorption peak of furfuryl alcohol. The intensity of this peak is slightly weaker than that of the furfuryl alcohol spectrum, corresponding to the number of waves of strong absorption peaks.

Furthermore, the absorption peaks of furfuryl alcohol due to the furan ring at approximately 1,590 cm^−1^ and 1,600 cm^−1^ manifest corresponding absorptions on the aromatic DRB-KOH. In contrast, no vibration of the absorption peak is observed on the biochar without adsorbed spice. This observation suggests that furfuryl alcohol has been adsorbed onto the DRB (Sayğılı and Güzel, 2016; Huang et al., 2020).

Thermogravimetric (TG) was employed to investigate the pyrolysis behavior of the adsorbent materials and the release of spices. The peak position and magnitude of the differential thermal gravity (DTG) curve can more accurately reflect the thermal decomposition behavior and thermal stability of the sample. Figure 2C presents the TG and DTG plots of DRB, DRB-KOH, DRB-HNO_3_, and DRB-H_2_SO_4_/HNO_3_ adsorbents.

Among them, DRB-KOH exhibits two primary mass loss phases. In the temperature range of 30°C–523°C, a weight loss of 32% is observed, attributed to the evaporation of physisorbed species on the microporous surface. The second stage, occurring at 523°C–730°C, accounts for a weight loss of 67.2%, representing the combustion of carbon material and the release of volatile decomposition products.

DRB-HNO_3_ displays two stages of mass loss; the main temperature range of 30°C–443°C witnesses a weight loss of 28.5%. Subsequently, at 443°C–609°C, a weight loss of 61.83% is recorded, characterized by rapid weight loss due to the evacuation of the sample following concentrated nitric acid treatment, particularly affecting large pores and sheet-like crevices, rendering the structure susceptible to high-temperature collapse and pyrolysis.

DRB-H_2_SO_4_/HNO_3_ undergoes four stages of mass loss. The primary temperature range spans 30°C–149°C, with a weight loss of 3.31%, attributed to surface flavoring dissipation and slow weight loss. This is followed by stages at 149°C–185°C (weight loss: 5.84%), 185°C–449°C (weight loss: 21.75%), and 449°C–626°C (weight loss: 64.56%), representing high-temperature pyrolysis causing structural damage and rapid weight loss.

In Figure 2D, the thermal decomposition of furfuryl alcohol is characterized by a single mass loss phase, occurring within the main temperature range of 50°C–160°C, where furfuryl alcohol decomposes vigorously, resulting in a weight loss of 90.52% when the temperature reaches 160°C.

DRB experiences a weight loss of only 9.78% when heated to 400°C and 60% when heated to 608°C, indicating slow decomposition and structural stability. However, compared to furfuryl alcohol and DRB, the thermal decomposition of DRB after spice adsorption can be divided into three stages of mass loss decomposition stage (33°C–326°C), characterized by relatively stable and slow decomposition, with a mass loss of about 8%, primarily due to water evaporation from the charcoal material and decomposition of spices on its surface. Gradual decomposition stage (326°C–609°C) is characterized by a noticeable weight loss as the temperature increases. During this phase, the biochar material decomposes gradually, releasing CO_2_ and water vapor due to high temperatures, resulting in structural damage and gradual release of the adsorbed spices (Ahmad et al., 2023; Sakai et al., 2017). By comparing the adsorbed biochar material with the adsorbed spices, it can be found that the fastest rate of weight loss was found at 123°C.

Rapid decomposition stage (above 609°C): marked by a faster mass loss than the adsorbed spices, with a weight loss of approximately 64.89% within the temperature range of 326°C–609°C. This suggests successful encapsulation of the spices within the biochar pores, achieving a slow-release effect.

### 3.4 Single-factor experiment of DRB

The optimization of calcination temperature and time for dandelion extract residue was conducted using adsorption rate and adsorption amount as evaluation criteria. Supplementary Figures 3, 4 illustrates the adsorption properties of biochar at various calcination temperatures (400, 500, 600, 700°C). As the calcination temperature increases, the decomposition of cellulose, hemicellulose, and lignin in tobacco straw generates a significant amount of water vapor, tar, and other byproducts (Bezerra et al., 2018; Charmas et al., 2021). The yield of biochar obtained at different calcination temperatures is presented in Supplementary Table 2. Notably, as the calcination temperature rises, the biochar yield decreases, indicating complete pyrolysis of cellulose, lignin, and other components in DRs, resulting in increased carbonization degree.

Supplementary Figure 3 shows that the adsorption rate initially increases and then decreases with rising calcination temperature. The carbon material obtained at 600°C exhibits the highest adsorption rate of 29.67% for spices, with an adsorption capacity of 123.89 μg/mL. Thus, 600°C is identified as the optimal calcination temperature for further optimization of the calcination time.

Supplementary Figure 4 illustrates the impact of different calcination times (1, 2, 4, 6, and 8 h) on the adsorption rate of spices for dandelion root biochar. As indicated in Supplementary Table 3, biochar yield remains relatively stable with increasing calcination time. However, excessive calcination time may lead to pore size deformation, decreasing spice adsorption rates. It is evident from Supplementary Figure 4 that the adsorption rate initially rises and then declines with prolonged calcination time. Notably, the carbon material obtained by maintaining a temperature of 600°C for 4 h exhibits the highest adsorption rate of 45.75%, with an adsorption amount of 186.28 mg/g.

Furthermore, as depicted in Supplementary Figure 5, significant differences in the adsorption rate of DRB are observed due to different adsorption methods, with ultrasound-assisted adsorption resulting in a 51.41% adsorption rate. Thus, ultrasound emerges as the most effective adsorption method.

In Supplementary Figure 6, the adsorption rate of DRB exhibits a trend of initial increase followed by a decrease with increasing adsorption time. The maximum adsorption rate of 51.41% is achieved at an adsorption time of 1 h, beyond which the adsorption rate diminishes. Thus, 1 h is identified as the optimum adsorption time.

Supplementary Figure 7 demonstrates that the adsorption rate of spice initially increases and then levels off with increasing adsorbent dosage. The maximum adsorption rate of 84.38% is attained when 200 mg of adsorbent is utilized. Subsequently, no significant increase in the adsorption rate is observed, and further increases in the adsorbent dosage are observed. Hence, 200 mg of adsorbent is deemed the optimum adsorption dosage.

As depicted in Supplementary Figure 8, with increasing spice concentration, the adsorption rate of the DRB first increases and then decreases. The maximum adsorption rate of 89.58% is reached when the concentration of adsorbed spice is 11.35 μg/mL. As the concentration of spice increases, the adsorption sites on the surface of the biochar material become saturated, leading to a decline in the adsorption rate. Therefore, 11.35 μg/mL is identified as the optimum concentration of adsorbed spice for optimizing the preparation conditions of DRB.

### 3.5 Adsorption and sustained-release kinetics

#### 3.5.1 Adsorption dynamics

**FIGURE 3:**
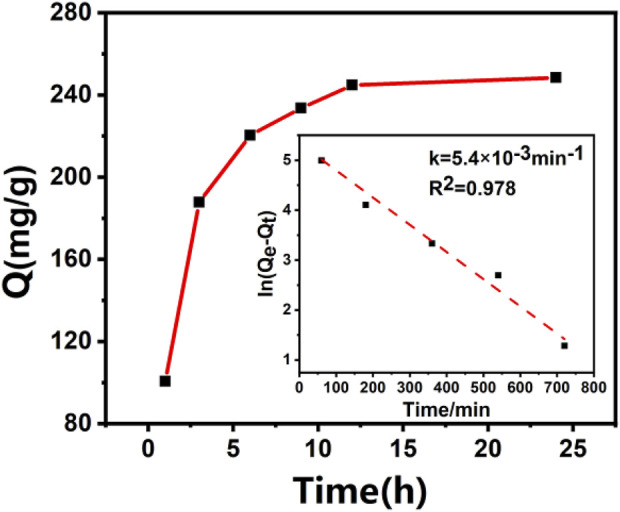
Adsorption kinetics of furfuryl alcohol on DRB-KOH.

Figure 3 is the adsorption kinetic curve and fitting kinetic constant of furfuryl alcohol on DRB-KOH material. The figure shows that the adsorption equilibrium for DRB-KOH is achieved after 12 h, with an adsorption equilibrium amount of 248.41 mg/g. The presence of the methylene group in furfuryl alcohol facilitates the donation of electrons to the aromatic ring, enabling furfuryl alcohol to form a stronger π-π interaction with DRB. This intensified π-π interaction may be the primary reason for the high adsorption capacity of furfuryl alcohol on the material. Consequently, furfuryl alcohol exhibits a faster adsorption rate and higher adsorption capacity on DRB materials (Jiho et al., 2020).

#### 3.5.2 Analysis of sustained-release curve under different temperature conditions

**FIGURE 4:**
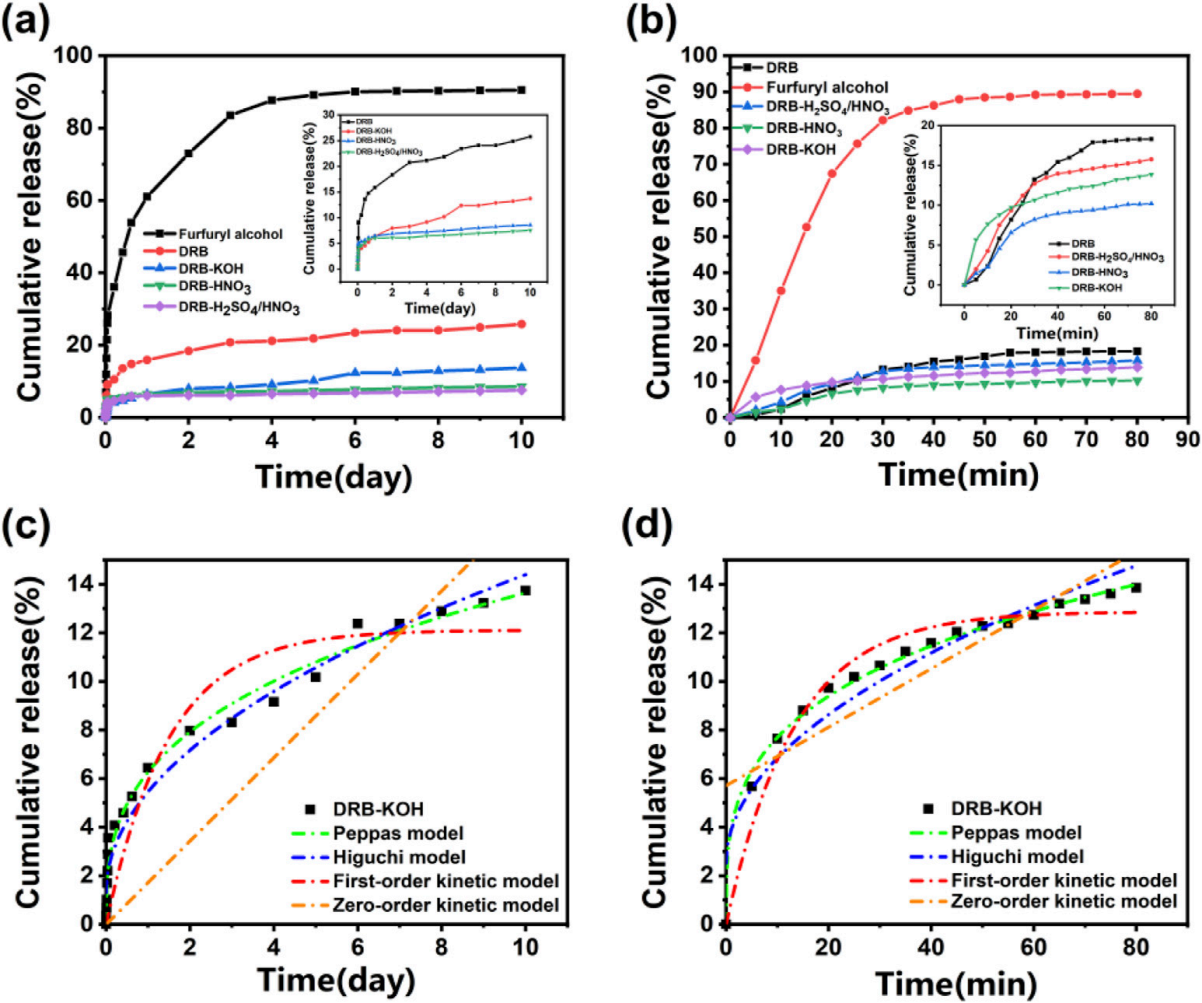
Spice release rate of spice and adsorbent at **(A)** 25°C and **(B)** 80°C; Release profiles of DRB-KOH at **(C)** 25°C and **(D)** 80°C.

Figure 4A shows the release curves of the spice and adsorbent at room temperature of 25°C, and Figure 4B shows the release curves of the spice and adsorbent at 80°C. As depicted in the figures, the release curves of both the spice and adsorbent samples under heating conditions at 25°C and 80°C exhibit a rapid initial weight loss followed by stabilization. Aromatic DRB, DRB-KOH, DRB-HNO_3_, DRB-H_2_SO_4_/HNO_3_) released an average of 9.83% over 2 days at 25°C, whereas furfuryl spice experienced a significantly higher release of 72.95% during the same period. Specifically, furfuryl spice lost 90.48% of its weight, while the aromatic DRB samples collectively lost an average of 14.52% of their weight after 10 days at 25°C.

Figure 4A illustrates a decrease in release from both acid-modified and base-modified adsorbents compared to unmodified DRB. Notably, DRB-KOH exhibited a decrease in release compared to DRB, while DRB-HNO_3_ and DRB-H_2_SO_4_/HNO_3_ showed an increase in release. This suggests that the pores of KOH-modified dandelion root biochar offer better protection for furfuryl alcohol flavoring, maintaining stability at room temperature for an extended period without significant damage.

Under heating conditions at 80°C (Figure 4B), the release of aromatic DRBs (DRB, DRB-KOH, DRB-HNO_3_, DRB-H_2_SO_4_/HNO_3_) underwent significant changes compared to room temperature conditions. Furfuryl alcohol reached release equilibrium at 45 min, with a release rate of 87.90%, while DRB approached stabilization at 55 min, with 17.89% released. Notably, DRB-KOH reached stabilization earlier than DRB, DRB-HNO_3_, and DRB-H_2_SO_4_/HNO_3_, releasing 10.65% at 30 min. This observation further confirmed the superior stability of alkali-modified dandelion root biochar under high-temperature conditions.

#### 3.5.3 Slow-release mechanism at different temperatures

To further investigate the release behavior of spice adsorbents at different temperatures, zero-order kinetics, first-order kinetics, the Higuchi model, and the Peppas model were employed to fit the release curves of spices at varying temperatures. The results, presented in Table 2 and Figure 4C, indicate that at 25°C, the release curve of the DRB adsorbent exhibited the highest degree of fitting with the Peppas model, yielding an R^2^ value of 0.948. Conversely, presented in Table 2 and Figure 4D, at 80°C, the release curve demonstrated the highest degree of fitting with the first-order kinetic model, yielding an R^2^ value of 0.969. These fitting outcomes suggest that the release and diffusion kinetics of the spice adsorbent can be effectively described by the Peppas model at lower temperatures and by the first-order kinetic model at higher temperatures.

**TABLE 2:** Release kinetics model of adsorbent at 25°C and 80°C.

Temperature	Adsorbent	Zero-order kinetic model		First-order kinetic model		Higuchi model		Peppas model	
		Equations	*R* ^2^	Equations	*R* ^2^	Equations	*R* ^2^	Equations	*R* ^2^
25°C	DRB	Y = 3.39x	0.193	Y = 22.23 (1-e^−2.52x^)	0.873	Y = 7.40 × ^1/2^ + 5.01	0.877	Y = 14.66 × ^0.26^	0.948
	DRB-HNO_3_	Y = 0.61x + 3.76	0.587	Y = 7.20 (1-e^−20.67x^)	0.908	Y = 2.07 × ^1/2^ + 2.88	0.751	Y = 5.94 × ^0.17^	0.919
	DRB-H_2_SO_4_/HNO_3_	Y = 0.52x + 3.35	0.574	Y = 6.39 (1-e^−18.90x^)	0.925	Y = 2.24 × ^1/2^ + 0.17	0.928	Y = 1.81 × ^2.57^	0.748
	DRB-KOH	Y = 1.72x	0.651	Y = 12.1141 (1-e^−0.67x^)	0.886	Y = 4.14 × ^1/2^ + 1.32	0.972	Y = 6.27 × ^0.34^	0.987
80°C	DRB	Y = 0.29x + 1.89	0.817	Y = 22.48 (1-e^−0.03x^)	0.969	Y = 2.57 × ^1/2^–2.62	0.933	Y = 1.32 × ^0.63^	0.930
	DRB-HNO_3_	Y = 0.17x	0.580	Y = 10.67 (1-e^−0.04x^)	0.978	Y = 1.28 × ^1/2^–0.05	0.918	Y = 1.45 × ^0.47^	0.921
	DRB-H_2_SO_4_/HNO_3_	Y = 0.26x	0.571	Y = 16.33 (1-e^−0.04x^)	0.986	Y = 1.95 × ^1/2^–0.01	0.925	Y = 2.28 × ^0.46^	0.928
	DRB-KOH	Y = 0.12x + 5.71	0.748	Y = 12.87 (1-e^−0.08x^)	0.959	Y = 1.37 × ^1/2^–2.51	0.934	Y = 3.97 × ^0.29^	0.996

According to the Peppas model, the sustained release of the spice adsorbent was analyzed. The “n” value in the Peppas model indicates the release mechanism of the spice within the adsorbent. When n ≤ 0.45, the release follows Fickian diffusion; when 0.45 ≤ n ≤ 0.85, it indicates non-Fickian diffusion; when n ≥ 0.85, the release mechanism is characterized by matrix dissolution (Dima et al., 2016). From Table 2, the fitting result of the Peppas model for DRB-KOH at 25°C yields n = 0.34, which is less than 0.45, suggesting that the slow-release mechanism of the adsorbent at 25°C follows Fickian diffusion.

On the other hand, the slow-release mechanism of DRB, DRB-HNO_3_, and DRB-H_2_SO_4_/HNO_3_ adsorbents at 80°C aligns more closely with the first-order kinetic equation. This can be attributed to the continuous volatilization of spices under high-temperature conditions, reducing the spices contained within the adsorbent and consequently decreasing the internal driving force for volatilization.

## 4 Conclusion

This comprehensive study on dandelion roots utilized multiple analytical methods to establish a framework for sustainable green development, exploring the potential of zero-waste multistage utilization of DRs. The extracts of DRs, rich in biologically active components, are suitable for chemical raw materials, biomedical agents, and food additives, especially due to their antioxidant properties. Furthermore, through pyrolysis and acid or alkali treatment, DRs can be converted into biochar and adsorbent materials for spice adsorption, leading to the development of a new sustained-release material. Release property investigations showed that the fragrance release follows the first-order kinetic model and Peppas model, indicating high fragrance retention. These results highlight DRs’ temperature stability and potential in various fragrance-related industries, overall promoting sustainable practices and showcasing the multifaceted potential of DRs.

## Data Availability

The original contributions presented in the study are included in the article/Supplementary Material, further inquiries can be directed to the corresponding author.
