# Supplementary material for: Zero-waste multistage utilization of dandelion root
Source: Front Chem. 2024 Aug 23;12:1457813. doi: 10.3389/fchem.2024.1457813 (PMC11377288; doi:10.3389/fchem.2024.1457813)
Supplement: Supplementary file 1 [file DataSheet1.PDF]

Supporting information

Zero waste multistage utilization of Dandelion root

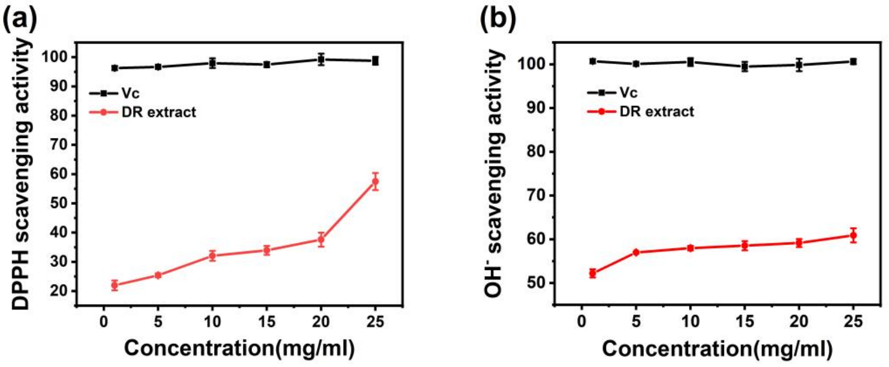

Fig. S1(a) Scavenging capacity of DPPH by extracts of different mass concentrations; (b) is the scavenging capacity of hydroxyl radicals by extracts of different mass concentrations

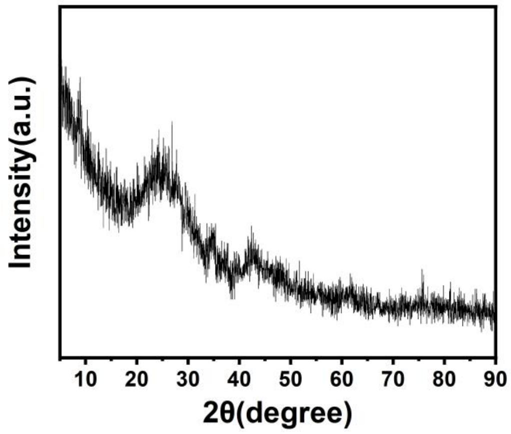

Fig. S2 XRD pattern of DRB

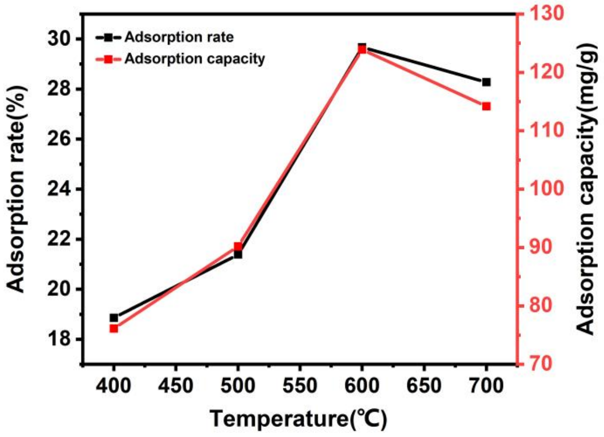

Fig. S3 Effect of calcination temperatures on the adsorption rate of biochar

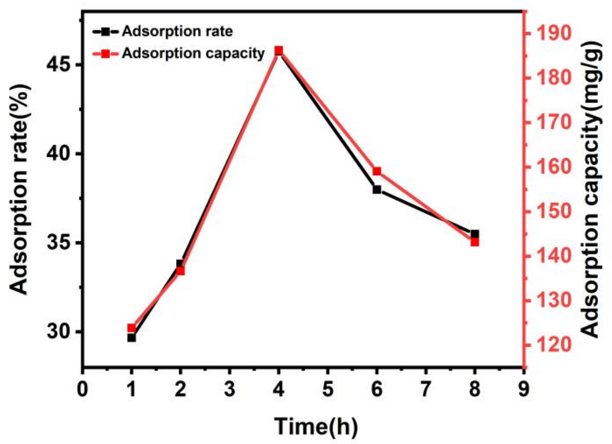

Fig. S4 Effect of calcination time on the adsorption rate of biochar

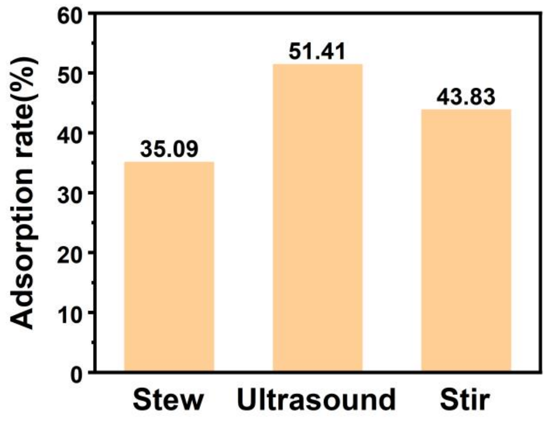

Fig. S5 Effect of adsorption methods on biochar adsorption rate

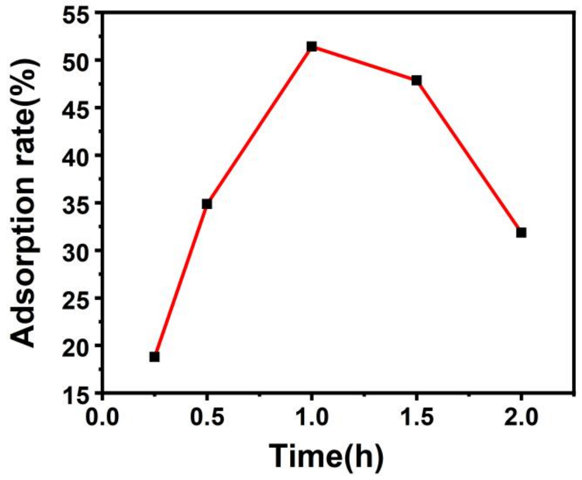

Fig. S6 Effect of adsorption time on biochar adsorption rate

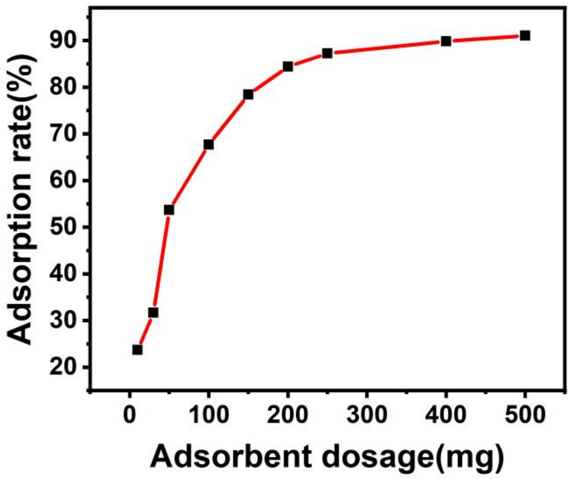

Fig. S7 Effect of adsorbent dosage on biochar adsorption rate

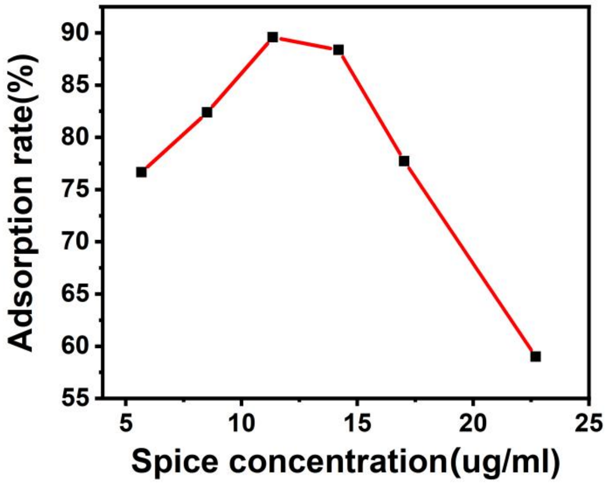

Fig. S8 Effect of spice concentration on biochar adsorption rate

Tab. S1 Classification of volatile constituents of dandelion root

Types	Quantities
Heterocyclic	6
Ester	11
Phenol	7
Aldehyde	4
Alkane	4
Ketone	12
Acid	5
Acylamide	1

Tab. S2 Biochar yield at different calcination temperatures

Temperature(℃)	Time(h)	Yield(%)
400	1	42.32

500	1	36.32
600	1	34.78
700	1	32.09
600	2	30.38
600	4	32.82
600	6	34.83
600	8	30.58
